# Thrombocytopenia Due to Direct Oral Anticoagulation and Low-Molecular-Weight Heparin

**DOI:** 10.7759/cureus.18757

**Published:** 2021-10-13

**Authors:** Madeline Tucker, Akhil Padarti

**Affiliations:** 1 Neurology, University of South Alabama College of Medicine, Mobile, USA

**Keywords:** heparin-induced thrombocytopenia, dural venous sinus thrombosis, pulmonary embolism, hemorrhagic cerebral infarction, direct oral anticoagulation

## Abstract

Direct oral anticoagulants (DOACs) are becoming increasingly prevalent in the general population for anticoagulation. However, rare adverse effects from these medications are still being discovered. Thrombocytopenia has previously been reported with these medications, but its clinical significance is still unknown. We present a patient who developed thrombocytopenia from apixaban and who subsequently developed a severe presentation of heparin-induced thrombocytopenia (HIT) from enoxaparin. This raises the possibility that thrombocytopenia from oral anticoagulants increases the likelihood of the development of heparin-induced thrombocytopenia.

## Introduction

Anticoagulation medications are extensively used for the treatment and prevention of arterial and venous thrombosis, including atrial fibrillation and pulmonary embolism. Historically, heparin and warfarin have long been the most commonly used anticoagulants; however, with the newer development of direct oral anticoagulants (DOACs), the therapeutic realm of thromboprophylaxis and treatment has greatly expanded. Since 2010, DOACs have been introduced into the market and are currently the preferred choice of anticoagulation for a majority of patients due to the ease of administration [1]. Since DOACs have only been utilized for a short period of time, the rare side effects are not as well studied as those of heparin or warfarin.

Heparin-induced thrombocytopenia (HIT) is a complication of heparin that occurs in 5% of patients exposed to unfractionated heparin and has even been seen in those exposed to low-molecular-weight heparin [2]. It typically presents after an exposure period of greater than four days and is caused by an autoantibody formation to platelet factor four (PF4), which leads to an immune complex formation between heparin molecules and platelets [3]. HIT causes widespread platelet activation and aggregation, which produces paradoxical thrombosis in up to 50% of patients [4]. The adverse effects of DOACs have not been fully expounded due to their novelty. Apixaban is a direct factor Xa inhibitor that possessed side effects of thrombocytopenia in the initial clinical trials [5,6]. Although these findings were not statistically significant, there have been a few case reports of DOAC-induced thrombocytopenia (DOAC-IT), suggesting that it is a true medication side effect [7-10]. The severity and clinical relevance of thrombocytopenia remain unknown.

The treatment for HIT and DOAC-IT is twofold: to remove the offending agent and to transition the patient to a different anticoagulation agent, i.e., argatroban or warfarin. These patients usually require long-term anticoagulation, particularly in the setting of severe hypercoagulability. It is often not practical to remove all anticoagulants completely. However, there have been no reports of the emergence of thrombocytopenia due to a secondary anticoagulant. Here, we report a case of apixaban-induced thrombocytopenia and subsequent heparin-induced thrombocytopenia, leading to severe hypercoagulability and multiorgan damage. The stepwise development of both hypercoagulable conditions may have had a synergistic effect resulting in multiorgan thrombosis and a life-threatening presentation.

## Case presentation

Our patient is a 69-year-old Caucasian female who underwent an elective left total knee replacement. There were no intraoperative or postoperative complications with documented stability in her routine laboratory work (Table 1). On postoperative day (POD) 2, the patient was discharged on Eliquis 2.5 mg BID for deep vein thrombosis (DVT) prophylaxis. On postoperative day 10, the patient presented to the ED for right flank pain and emesis. She was readmitted to the hospital and diagnosed with a postoperative ileus and urinary tract infection. At the time of the second admission, our patient had developed thrombocytopenia and an elevated D-dimer (Table 1). No workup for her thrombocytopenia was performed at this second hospitalization. During the second hospitalization, she received enoxaparin for routine DVT prophylaxis. She spent four days in the hospital for supportive care and IV antibiotics. At discharge, all anticoagulants were discontinued.

**Table 1:** Routine Laboratory Work Throughout Hospitalization Routine coagulation laboratory work obtained during the three hospitalizations is shown. Pre-OP and POD 2 laboratory results were obtained during the first elective hospitalization. POD 10 and POD 12 laboratory results were from the second hospitalization. POD 18 to POD 44 laboratory results were from the third hospitalization. (-) is any laboratory values less than the lower limit of normal, and (+) is any laboratory values higher than the upper limit of normal. Abbreviations: POD, postoperative day; INR, international normalized ratio.

	Pre-OP	POD 2	POD 10	POD 12	POD 18	POD 27	POD 44
Platelets	189 x 10^3^/mcL	153 x 10^3^/mcL	71 x 10^3^/mcL (-)	62 x 10^3^/mcL (-)	39 x 10^3^/mcL (-)	71 x 10^3^/mcL (-)	98 x 10^3^/mcL (-)
INR	1.01	-	-	-	1.25 (+)	1.60 (+)	2.89 (+)
D-Dimer	-	-	-	18.06 mcg/mL (+)	-	17.44 mcg/mL (+)	-

**Figure 1:**
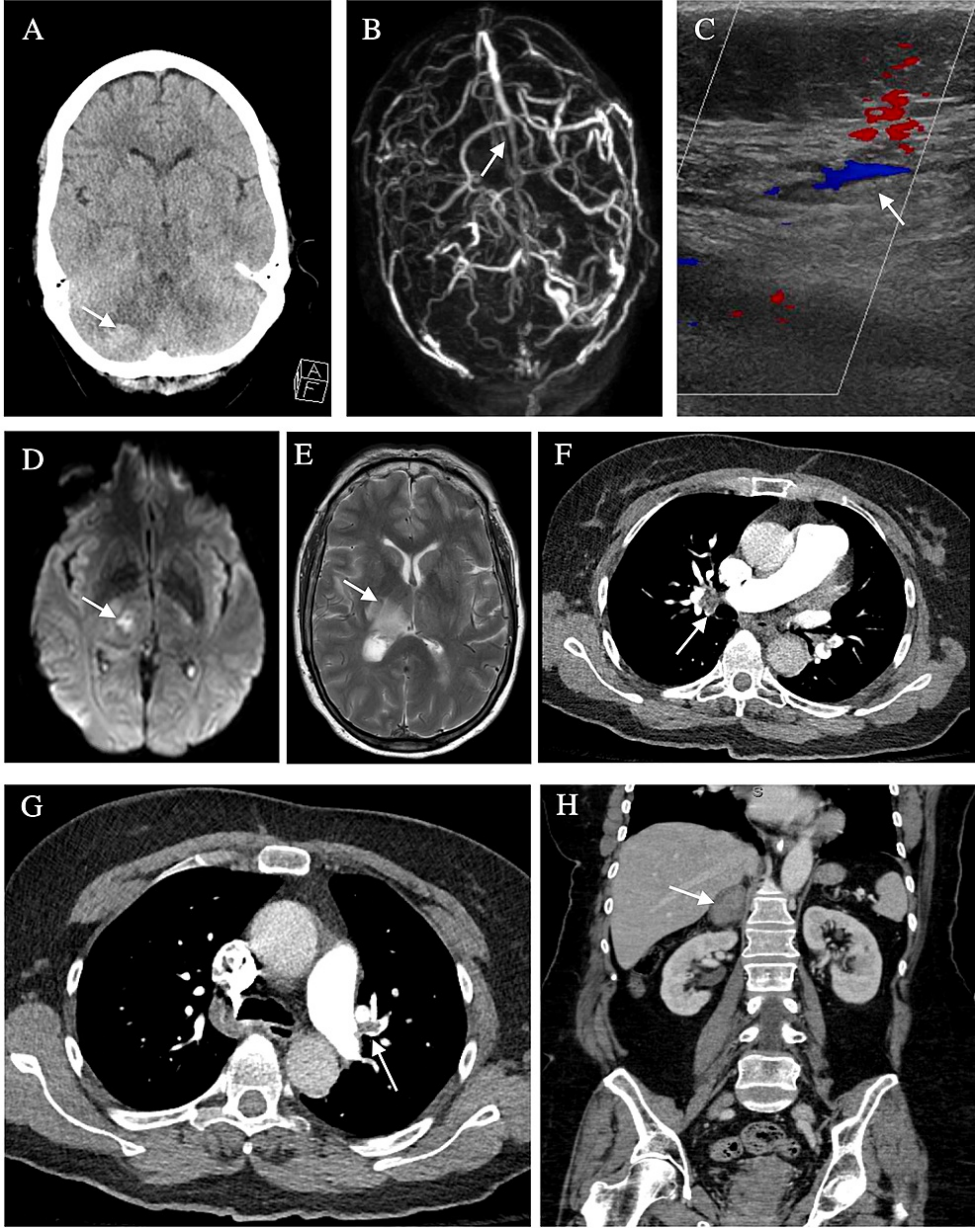
Radiographic Images of Multiorgan Thrombosis and Hemorrhage Select radiographic imaging of the patient's workup during the third hospitalization. Arrows point to pathology where appropriate. A) Axial CT brain showing right cerebellar hemorrhage. B) 3D reconstruction of the MRV brain showing superior sagittal thrombosis and right transverse sinus thrombosis. C) Lower extremity venous doppler of the left posterior tibial vein showing age-indeterminate venous thrombosis. D) MRI brain DWI sequence showing diffusion restriction of the right cerebral peduncle. E) MRI brain T2 sequence showing right thalamic hemorrhage. F) Axial CTA chest showing right segmental pulmonary embolism. G) Axial CTA chest showing left pulmonary artery embolism. H) Coronal CT abdomen and pelvis with contrast showing large subacute right adrenal hematoma.

After discharge, the patient had a waxing and waning mental state, progressive weakness, and headache. On POD 16, she presented to the hospital with sharp right-sided chest pain. Her physical examination showed a focal neurological weakness of the left face, arm, and leg. Laboratory work showed thrombocytopenia (Table 1). Her initial imaging was significant for multiorgan thrombosis and hemorrhage (Figure 1). CT head showed a right cerebellar hemorrhage (Figure 1A). MRV brain showed multiple venous sinus thromboses in the right sigmoid sinus, right transverse venous sinus, posterior superior sagittal sinus, and straight sinus (Figure 1B). Venous dopplers showed a left posterior tibial venous thrombus (Figure 1C). MRI brain showed a right thalamic hemorrhage and an acute infarct of the right cerebral peduncle (Figure 1D, 1E). CTA chest showed left pulmonary artery embolism and bilateral segmental/subsegmental pulmonary emboli (Figure 1F, 1G). CT abdomen showed a subacute right adrenal hematoma (Figure 1H).

The patient was admitted to the ICU and initially started on a heparin infusion for acute thrombosis, which resulted in further worsening of her thrombocytopenia. Laboratory workup results for thrombocytopenia were obtained (Table 2). Heparin infusion was subsequently discontinued. A serotonin release assay and heparin/PF4 complex were positive, confirming the diagnosis of HIT (Table 2). Our patient was switched to argatroban but continued to have persistent thrombocytopenia. She received three units of platelet transfusions during this hospitalization. Blood cultures were drawn and remained negative; no antibiotics were administered. After five days of stability on argatroban, the patient was transferred from the ICU and continued to improve cognitively and functionally. Her thrombocytopenia stabilized with a platelet count of 88-89 × 10^3^/mcL. She was transitioned from an argatroban infusion to warfarin with INR at goal before she was ultimately discharged to an inpatient rehabilitation facility.

**Table 2:** Thrombocytopenia Laboratory Workup Results Select laboratory results from the thrombocytopenia workup are shown. The patient’s laboratory result is shown in column 2, and the normal laboratory ranges are shown in column 3. (-) is any laboratory values less than the lower limit of normal, and (+) is any laboratory values higher than the upper limit of normal.

	Patient’s Laboratory Values	Normal Laboratory Ranges
Fibrinogen	388 mg/dL	192–479 mg/dL
Antithrombin III	114%	80%–120%
Heparin/platelet factor 4 complex	Positive	Negative
Serotonin release assay	Positive	Negative
Protein C	166%	83%–168%
Protein S	119%	55%–123%
Factor VIII assay	193% (+)	60%–150%
Factor IX assay	223% (+)	60%–150%
Factor XI assay	145% (+)	60%–140%
Von Willebrand factor	286% (+)	51%–215%
Antiphospholipid Ab IgG	4 GPL	0–14 GPL
Cardiolipin IgG	2 GPL	0–14 GPL
B2-glycoprotein I IgG	0 SGU	0–20 SGU
Lupus anticoagulant	36 seconds	23–38 seconds

## Discussion

DOAC-induced thrombocytopenia

Apixaban is one of the newer oral anticoagulants that is a direct inhibitor of factor Xa. Although first approved in 2011, its indication of use has steadily increased. Amidst the growing widespread usage, certain newer side effects of apixaban have been detected. Thrombocytopenia was seen in <1% of treatment groups in the clinical trials for apixaban but was not significant from placebo [5,6]. Thrombocytopenia was also seen with other oral anticoagulants [9,10]. The mechanism of thrombocytopenia remains unknown. Possible mechanisms include immune-mediated destruction and bone marrow suppression; however, there is a paucity of literature corroborating this reaction [7,8]. This is possibly due to underrecognition of this side effect. Our patient received apixaban at discharge from her first elective hospitalization and developed thrombocytopenia seen on admission laboratory examinations for her second hospitalization. She had not received any other anticoagulation medications except for apixaban; therefore, the etiology of the thrombocytopenia was apixaban. She had sustained thrombocytopenia after discontinuing apixaban, suggesting that the mechanism of the thrombocytopenia may be bone marrow suppression rather than immune-mediated destruction. Because her clinical course was complicated by the development of HIT, this pattern of sustained thrombocytopenia is unique and not observed in previous case reports [7,8].

HIT due to enoxaparin

Heparin has long been implicated in the syndrome of antibody-mediated thrombocytopenia known as HIT [11]. Two types of HIT have been identified. Type 1 has a benign course with a transient decrease in platelets between days 1 and 4 of heparin administration, and type II results in a paradoxical prothrombotic state between days 5 and 15 [12]. Type II HIT may lead to a severe clinical presentation referred to as “catastrophic HIT” due to resulting multiorgan system damage [13]. Enoxaparin is low-molecular-weight heparin that has been implicated in the pathogenesis of HIT [14]. Our patient developed type II HIT seven days after the administration of enoxaparin during her second hospitalization. She had multiple known risk factors for HIT, i.e., urosepsis and orthopedic surgery [4]. Our patient’s calculated 4Ts score was 7, due to the platelet count fall of >50%/platelet nadir of >20, onset of symptoms on day 7, new thrombosis, and possible other causes of thrombocytopenia due to her apixaban use. This score is suggestive of a high probability (~64%) of HIT. Laboratory work showed that she was positive for HIT antibodies (Table 2). She developed widespread arterial and venous thrombosis and multiorgan hemorrhage as evident by her radiographic images. This is the second reported case of HIT from enoxaparin with thrombosis involving cerebral, pulmonary, abdominal, and lower extremity vessels [13]. Our patient presented with a severe case of HIT, likely due to a coexisting NOAC-IT from apixaban. The combination of both conditions may have a synergistic effect on the coagulation cascade.

Difficulty with subsequent anticoagulation

Patients with HIT usually require anticoagulation to mediate the thrombotic effects. Argatroban and bivalirudin are the two direct thrombin inhibitors that are FDA approved for this indication. However, there have been promising studies on the use of oral anticoagulants for HIT [15]. Oral anticoagulants have advantages over the other formulations, including the ease of medication administration, as both argatroban and bivalirudin are infusions. Additionally, DOACs do not need close INR monitoring, unlike warfarin. However, our patient developed HIT and DOAC-IT, sequentially raising the possibility that certain patients are at a predisposition to both reactions. For our patient, warfarin was the agent of anticoagulation at discharge due to socioeconomic factors, while argatroban was the bridging agent. Our patient had persistent thrombocytopenia even after the removal of both heparin and apixaban, and the exact etiology of this remains unclear. A majority of patients with HIT (65%) regain platelet levels within one week [16], and no case series are available for NOAC-IT. The possibility that the persistent thrombocytopenia is from the synergistic effect of NOAC-IT and HIT still exists, but further research is needed.

## Conclusions

With the increasing usage of newer oral anticoagulants, rare but serious side effects are emerging, including DOAC-induced thrombocytopenia. Unfortunately, little is known about the long-term side effects and implications of this condition. This case presentation showed a patient that developed HIT after DOAC-IT. It is unclear whether patients with DOAC-IT are at an increased risk of HIT, and more research needs to be performed in order to establish this correlation. Until then, these patients should be closely monitored for any life-threatening side effects.
